# Plasmids of Distinct IncK Lineages Show Compatible Phenotypes

**DOI:** 10.1128/AAC.01954-16

**Published:** 2017-02-23

**Authors:** Marta Rozwandowicz, Michael S. M. Brouwer, Aldert L. Zomer, Alex Bossers, Frank Harders, Dik J. Mevius, Jaap A. Wagenaar, Joost Hordijk

**Affiliations:** a Department of Infectious Diseases and Immunology, Faculty of Veterinary Medicine, Utrecht University, Utrecht, The Netherlands; b Wageningen Bioveterinary Research, Lelystad, The Netherlands

**Keywords:** IncK, plasmid, incompatibility

## Abstract

IncK plasmids are some of the main carriers of *bla*_CTX-M-14_ and *bla*_CMY-2_ genes and show high similarity to other plasmids belonging to the I complex, including IncB/O plasmids. Here, we studied the phylogenetic relationship of 37 newly sequenced IncK and IncB/O plasmids. We show that IncK plasmids can be divided into two compatible lineages named IncK1 and IncK2.

## TEXT

Antimicrobial resistance due to extended-spectrum beta-lactamases (ESBL) and AmpC beta-lactamases is a global problem. Among the most prevalent variants are *bla*_CMY-2_ and *bla*_CTX-M-14_, both of which are often carried on IncK plasmids from different sources (1–7). IncK plasmids are highly related to IncB/O, IncZ, and IncI plasmids, which all belong to the I complex (8). The compatibility of IncB/O plasmids was extensively studied previously (9). Knowledge on IncZ plasmids was limited until recently, when Moran et al. (10) showed that there are multiple variants of IncZ plasmids. To better understand the complexity of the I complex, the purpose of this study was to further investigate the phylogenetic relationship of IncK plasmids to IncB/O and to determine compatibility within and between potential IncK lineages.

Escherichia coli isolates from The Netherlands carrying *bla*_CMY-2_ or *bla*_CTX-M-14_ were selected from various ESBL studies performed within our laboratory. These were screened for IncK plasmids using previously described primers (11). Sequencing results were used to confirm the presence of an IncK replicon, as in several cases the PCR-based replicon typing (PBRT) gave an ambiguous result. Additionally, an IncB/O plasmid was added as an outgroup.

**FIG 1:**
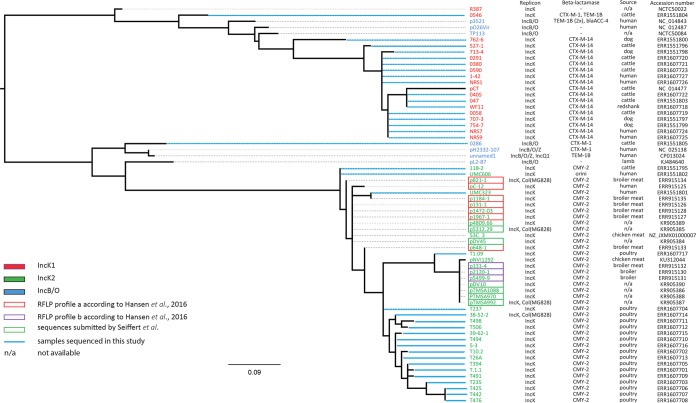
Phylogenetic tree of IncK and IncB/O plasmids.

For all experiments, IncK or IncB/O plasmids were transferred by either transformation or conjugation. For transformation, plasmids were isolated using the Wizard Plus SV kit (Promega) and transformed to E. coli DH10B ElectroMAX cells (Thermo Fisher Scientific), according to the manufacturer's instructions. Selection of transformants was performed on Luria-Bertani (LB) agar plates (Oxoid/Tritium) supplemented with 2 mg/liter cefotaxime (Sigma). Conjugation was performed as previously described (12) with exconjugants recovered on LB plates supplemented with 2 mg/liter cefotaxime and 75 mg/liter rifampin or 25 mg/liter chloramphenicol. Conjugation was confirmed by PCR on relevant targets. Thirty-six IncK and 1 IncB/O plasmid-carrying E. coli were subjected to whole-genome sequencing (WGS). WGS was performed on an Illumina MiSeq platform using 2 × 250-bp reads and a 300-bp insert size. Assembly was performed using SPAdes (13) with the default settings. Chromosomal contigs were removed by mapping against either DH10B, MG1655, or W3110 genome sequences using BLAST (14). The remaining plasmid contigs were annotated using Prokka (15). Screening for antimicrobial resistance genes was performed using ResFinder (16). Core and pan genome determination and whole-plasmid-based phylogeny were performed with Roary (17), using the nonparalog splitting method. A phylogenetic tree was constructed from both newly sequenced and downloaded sequences using FastTree (18). The phylogenetic tree was visualized using interactive tree of life (iTOL) (19). Plasmid contigs were submitted to the European nucleotide archive (http://www.ebi.ac.uk/ena) with the accession numbers listed in Fig. 1. Additional IncK and IncB/O plasmid sequences were obtained from the Wellcome Trust Sanger Institute (http://www.sanger.ac.uk/resources/downloads/plasmids/), GenBank, and the European nucleotide archive.

**FIG 2:**
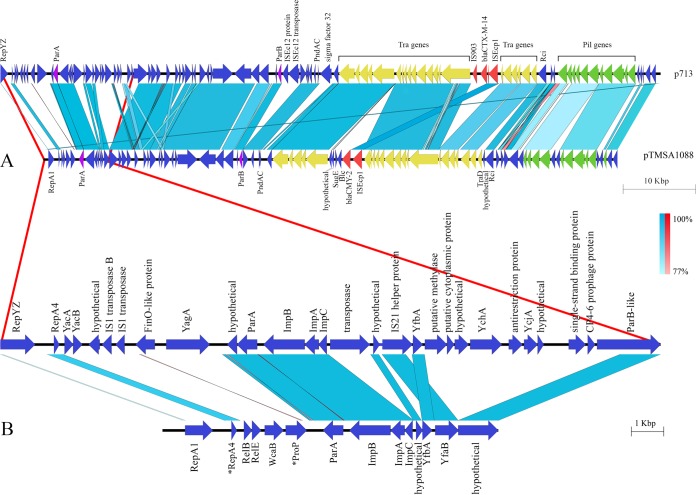
Comparison of the IncK1 and IncK2 plasmid sequences (A) with an additional focus on the highest variable region in the plasmid scaffolds (B). Asterisks indicate partial gene sequences.

Plasmid sizes ranged from 79,176 to 168,100 bp, with an average GC content of 52.6% and 104 coding sequences. The core genome of all IncK plasmids includes transfer and partition systems and the shufflon recombinase with the *pilV* gene. *bla*_CTX-M14_ was associated with IS*Ecp1* upstream and IS*903* downstream. In contrast, *bla*_CMY-2_-carrying plasmids lack IS*903*, except for one *bla*_CMY-2_- and the *bla*_CTX-M-1_-carrying plasmid, which both lacked IS*Ecp1* as well as IS*903*. IncK1 and IncK2 vary in the presence or absence of several genes (Fig. 2B), which may be caused by the presence of multiple insertion sequences.

Analysis of the phylogenetic tree based on the absence or presence of accessory genes of IncK and IncB/O plasmids (Fig. 1) revealed the presence of two major clusters containing IncK plasmids. The first cluster, designated IncK1, contains plasmids carrying *bla*_CTX-M-14_. IncK1 shows high similarity to the previously described pCT (20). The second cluster, IncK2, includes IncK plasmids carrying *bla*_CMY_ genes. Overall, IncK1 and IncK2 plasmids show high levels of homology (Fig. 2A). Additionally, Hansen et al. (7) presented two IncK plasmid restriction fragment length polymorphism (RFLP) types (SalI digested), which form two distinct clusters, both within the IncK2 lineage (Fig. 1).

Due to accumulation of single nucleotide polymorphisms (SNPs) in the target region of IncK1 plasmids, some typing results were difficult to reproduce using previously designed primers (11). Therefore, a new set of primers was designed (see Table S1 in the supplemental material). New and previously reported primers were mixed in a 1:1 ratio to a final concentration of 2.5 pmol/μl each. PCR was performed using GoTaq green master mix (Promega) with an annealing temperature of 63°C. Additionally, two pairs of primers, targeting RNAI and part of the *repY* gene, were designed to discriminate between the IncK1 and IncK2 plasmid lineages, using an annealing temperature of 55°C. Selected plasmids were subjected to incompatibility tests, which were performed via conjugation of two E. coli strains carrying different IncK or IncB/O plasmids. To determine the incompatibility of members of the IncK2 lineage, in which all plasmids carry the *bla*_CMY-2_ gene, an additional set of primers was used, targeting the *ssb* gene and IS*Ecp1* (see Table S1 in the supplemental material), which differed in the two selected IncK2 plasmids. Testing of exconjugants revealed that IncK1 and IncK2 plasmids were compatible (Table 1). In contrast, plasmids belonging to the same group, either IncK1 or IncK2, are incompatible. The compatibility of IncK1 and IncK2 was checked with IncB/O plasmids to confirm that neither of the lineages was a mistyped IncB/O plasmid. Additionally, stability of the plasmids from both lineages in one host was checked (method adapted from Jafar et al. [21]). Stability was determined as a percentage of colonies carrying both plasmids in the absence of selective pressure compared to that of those plated on selective agar. Fifty colonies carrying both IncK1 and IncK2 plasmids were plated on either LB agar or LB agar supplemented with appropriate antibiotics. Subculturing of all colonies was repeated every 24 h for 3 days. The presence of both plasmids was confirmed by PCR, targeting the RNAI and the resistance genes. After 72 h, 98% of the IncK1 plasmids were still present using selective agar and 100% using nonselective agar. The IncK2 plasmid showed 100% stability on both selective and nonselective agar.

**TABLE 1:** Incompatibility test results for mating pairs

Plasmid 1 (plasmid identification)	Plasmid 2 (plasmid identification)	Incompatibility result
IncK1 (p754)	IncK2 (p118)	Compatible
IncK1 (p0291)	IncK2 (p118)	Compatible
IncK1 (pWF11)	IncK1 (p527)	Incompatible
IncK2 (p39_62_1)	IncK2 (pT1.09)	Incompatible
IncK1 (p754)	IncK1 (p0546)	Incompatible
IncK2 (pT10.2)	IncK1 (p0546)	Compatible
IncK1 (p754)	IncB/O (p0289)	Compatible
IncK2 (p118)	IncB/O (p0289)	Compatible
IncK2 (p0291)	IncB/O (p0289)	Compatible

In conclusion, our results show the existence of two IncK plasmid lineages, which confirms the observation of Seiffert et al. (S. N. Seiffert, A. Carattoli, S. Schwendener, A. Endimiani, and V. Perreten, unpublished data), who submitted sequences of seven IncK2 plasmids to GenBank (Fig. 1) (accession no. KR905384 to KR905390). Within one lineage, plasmids are incompatible with each other, but they are compatible between lineages. The phylogenetic analysis could be possibly influenced by the geographical bias of the origin of plasmids included in this study. These findings should therefore be confirmed using an extended collection of plasmids from a more diverse background. The high similarity of IncK, IncB/O, and IncZ RNAI sequences, which are targets in the PBRT classification scheme, causes difficulties with typing. Further analysis is necessary to improve the tools that will allow better detection and discrimination of plasmids of the I complex.

## Supplementary Material

Supplemental material
