# Supplementary material for: Plasmids of Distinct IncK Lineages Show Compatible Phenotypes
Source: Antimicrob Agents Chemother. 2017 Feb 23;61(3):e01954-16. doi: 10.1128/AAC.01954-16 (PMC5328535; doi:10.1128/AAC.01954-16)
Supplement: Supplemental material [file supp_61_3_e01954-16__index.html]

Plasmids of Distinct IncK Lineages Show Compatible Phenotypes — Supplemental material 

# Plasmids of Distinct IncK Lineages Show Compatible Phenotypes

## Supplemental material

- Supplemental file 1 -

  Table S1

  PDF, 119K
