# Supplementary material for: Plasmids of Distinct IncK Lineages Show Compatible Phenotypes
Source: Antimicrob Agents Chemother. 2017 Feb 23;61(3):e01954-16. doi: 10.1128/AAC.01954-16 (PMC5328535; doi:10.1128/AAC.01954-16)
Supplement: Supplemental material [file AAC.01954-16_zac003175976s1.pdf]

Supplementary table S1 Primers used in this study

Primer name	Primer sequence	Target	Reference
K/B FW	gcggtccggaaagccagaaaac	RNAI	(1)
K RV	tctttcacgagcccgccaaa		(1)
K/B fv new	aggatccgggaagtcagaaaac	RNAI	this study
K rv new	tctttcacgatcccgccaaa		this study
K1fv	atcgtcaggatccgggaagtc	RNAI and *repY* gene	this study
K1rv	gagcgattgtgccgtgtatt		this study
K2fv	atgctcgcggtccggaaagcc	RNAI and *repY* gene	this study
K2rv	gtgccgtgcgttaatgcactgcaa		this study
CMY-2-F	atgatgaaaaaatcgttgctgc	bla_{CMY} gene	(2)
CMY-2-R	gcttttcaagaatgcgccagg		(2)
TEM-F	gcggaacccctatttg	bla_{TEM} gene	(3)
TEM-R	accaatgcttaatcagtgag		(3)
CTX-M-9-1F	tggtgacaaagagagtgcaacg	$bla_{CTX\text{-}M\text{-}group\text{-}9}$ genes	(4)
CTX-M-9-4R	tcacagcccttcggcgat		(4)
CTX-M-9_{792}F	ctattttacccagccgcagc	$bla_{CTX\text{-}M\text{-}14}$ gene	(5)
CTX-M-9_{1029}R	gttatggagccacggttgat		(5)
ssb fv	tggtgctgttcggcaagctc	*ssb gene*	this study
ssb rv	ccttacgtccacggcctttc		this study
ISEcp1A	gcaggtctttttctgctcc	IS*Ecp1B* transposase	(6)
ISEcp1B	tttccgcagcaccgtttgc		(6)

References

1. **Carattoli, A, Bertini, A, Villa, L, Falbo, V, Hopkins, KL, Threlfall, EJ.** 2005. Identification of plasmids by PCR-based replicon typing. J. Microbiol. Methods. **63:**219-228.

2. **Dierikx, C, van Essen-Zandbergen, A, Veldman, K, Smith, H, Mevius, D.** 2010. Increased detection of extended spectrum beta-lactamase producing *Salmonella enterica* and *Escherichia coli* isolates from poultry. Vet. Microbiol. **145:**273-278

3. **Olesen, I, Hasman, H, Aarestrup, FM.** 2004. Prevalence of beta-lactamases among ampicillin-resistant *Escherichia coli* and *Salmonella* isolated from food animals in Denmark. Microb. Drug Resist. **10:**334-340.

4. **Paauw, A, Fluit, AC, Verhoef, J, leverstein-van hall, MA.** 2006. Enterobacter cloacae outbreak and emergence of quinolone resistance gene in Dutch hospital. Emerg. Infect. Dis. **12**:807-812

5. **Dierikx, CM, van Duijkeren, E, Schoormans, AHW, van Essen-Zandbergen, A, Veldman, K, Kant, A, Huijsdens, XW, van der Zwaluw, K, Wagenaar, JA, Mevius, DJ.** 2012. Occurence and characteristics of extended-spectrum-ß-lactamase- and AmpC-producing clinical isolates derived from companion animals and horses. J Antimicrob Chemother. **67**:1368-74

6. **Poirel, L, Decousser, JW, Nordmann, P.** 2003. Insertion sequence IS*Ecp1B* is involved in expression and mobilization of a $bla_{CTX\text{-}M}$ beta-lactamase gene. Antimicrob. Agents Chemother. **47:**2938-2945.
